# Diabetes Mellitus as an Integrated Microbiome, Immune, and Metabolic Disorder with Clinical Implications for Multisystem Complications and Public Health

**DOI:** 10.3390/jcm15051788

**Published:** 2026-02-27

**Authors:** Ayman Elbehiry, Eman Marzouk, Fahad A. Alhumaydhi, Adil Abalkhail

**Affiliations:** 1 Department of Public Health, College of Applied Medical Sciences, Qassim University, P.O. Box 6666, Buraydah 51452, Saudi Arabia; ar.elbehiry@qu.edu.sa (A.E.); e.marzouk@qu.edu.sa (E.M.); 2 Department of Medical Laboratories, College of Applied Medical Sciences, Qassim University, P.O. Box 6666, Buraydah 51452, Saudi Arabia; f.alhumaydhi@qu.edu.sa

**Keywords:** diabetes mellitus, gut microbiome, immune regulation, metabolic dysfunction, diabetic complications, public health, health promotion, integrated care models

## Abstract

Diabetes mellitus is one of the most common health problems worldwide; however, increased blood glucose alone cannot adequately explain its pathophysiology. Although high blood glucose is a defining feature, evidence increasingly proves that diabetes arises from systemic disturbances involving the gut microbiome, immune system, and metabolic control. From this perspective, diabetes can be viewed as a systemic condition shaped by the dynamic interactions between the gut microbiome, the immune system, and metabolic pathways. Alterations in gut microbiome composition and function can influence nutrient metabolism, microbial metabolite production, bile acid signaling, and intestinal barrier integrity. Any damage of the gut barrier allows movement of microbiome-derived molecules that activate innate immune pathways and provoke chronic low-grade inflammation. This inflammatory state interferes with insulin signaling, contributes to immune maladaptation, and exacerbates metabolic dysfunction. Over time, these processes contribute to the advance of multisystem complications, including cardiovascular disease, diabetic nephropathy, neuropathy with cognitive impairment, delayed wound healing, and increased susceptibility to infection. The review also integrates environmental and public health factors, demonstrating how diet, antibiotic exposure, circadian disruption, and social conditions shape the microbiome, immune function, metabolic regulation, and disease risk across the life course. By bringing together clinical, experimental, and population-based evidence, this review illustrates the limitations of care models that concentrate only on glucose. It also points out how integrated approaches targeting the microbiome, immune system, and metabolic pathways can improve diabetes prevention, management, and guide future research.

## 1. Introduction

Diabetes represents one of the leading causes of death and disability worldwide, and affects people regardless of country, age group, or sex [1]. Hundreds of millions of adults worldwide live with diabetes, with recent large-scale analyses estimating the total may exceed 800 million when various diagnostic methods are considered. Despite variations in data sources and methods, all indicate a significant and growing global burden [2,3]. Diabetes consequences extend beyond blood glucose dysregulation, significantly contributing to cardiovascular disease, kidney failure, vision loss, limb amputation, and premature death. These complications heavily burden healthcare systems and families, particularly in low- and middle-income countries with limited access to timely diagnosis and ongoing care [1,4].

Clinical practice and research have traditionally emphasized glycemic indicators and organ-specific outcomes, placing glycemic control at the core of diabetes management and enabling improved short-term metabolic control [5,6]. Nevertheless, similar levels of glycemia do not reliably predict clinical outcomes. Individuals with comparable glucose profiles often experience markedly different disease courses and complication risks [4,7], underscoring the role of mechanisms beyond glucose regulation in disease progression.

Previous studies have emphasized important links between the gut microbiota and metabolic features of diabetes. Nonetheless, they have not fully incorporated microbial, immune, and metabolic pathways within a single biological framework [8,9]. Bibliometric analyses display rapid growth in diabetes microbiome research over the past few decades, with the majority of investigations concentrated on metabolic homeostasis. These trends underscore the need for more integrated models that link metabolic regulation with immune and microbial processes [10].

This review addresses this need by presenting a unified framework that links microbiome biology, immune dysregulation, metabolic dysfunction, and public health determinants. Rather than treating these domains in isolation, it links mechanistic evidence across molecular, systemic, and population levels. This perspective connects gut barrier disruption, immune activation, multisystem complications, and environmental drivers within a single coherent model.

Recent investigations suggest that the gut microbiome interacts with both innate and adaptive immune responses to influence metabolic health. Findings from human studies and experimental models illustrate that microbial communities shape nutrient handling and produce metabolites that modulate host signaling. These processes also support intestinal barrier function and regulate immune activity at local and systemic levels [11,12].

Impairment of the intestinal barrier allows microbial products to enter the circulation and increase systemic inflammatory tone. Persistent low-grade inflammation can then disrupt insulin signaling and other metabolic pathways. This process links altered microbial signals to insulin resistance and inflammatory disease states [12,13]. Immune maladaptation in diabetes happens through numerous mechanisms. Loss of immune tolerance contributes to autoimmune beta cell injury, while impaired innate and adaptive responses increase susceptibility to infection and delay tissue repair. These disturbances produce feedback loops that deteriorate metabolic control and clinical consequences [14,15].

Understanding diabetes as an integrated disorder of the microbiome, immune system, and metabolism helps explain key clinical observations. It clarifies why environmental and social changes influence disease patterns across populations and why lifestyle factors, medications, and early-life exposures have lasting effects on risk. This perspective also expands opportunities for prevention and treatment beyond glucose lowering alone [16,17].

Given the growing burden of diabetes and the accumulating mechanistic evidence, a systems-level framework is timely. This review adopts such an approach to synthesize clinical and translational findings across disciplines. It outlines pathways linking microbial and immune states to multisystem complications, highlights clinical and public health implications, and identifies areas of established evidence as well as key gaps for future study.

Despite rapid development in microbiome and immunometabolic research, significant gaps remain. Numerous studies investigate microbial, immune, or metabolic pathways in isolation, with limited incorporation across systems or levels of evidence. Associations between mechanistic findings, multisystem complications, and public health determinants are also inconsistently addressed.

This narrative review is based mainly on peer reviewed clinical, experimental, and population-based studies relevant to the biology of diabetes. The literature was documented through searches of PubMed, Scopus, and Web of Science. Priority was given to studies published over the past few decades. We concentrated on systematic reviews, meta-analyses, large observational studies, and key experimental work with clear relevance to diabetes. Articles were selected based on their contribution to understanding interactions between microbial function, immune regulation, metabolic processes, and public health determinants. This approach was utilized to support a focused and integrative synthesis rather than an exhaustive systematic review.

**Figure 1:**
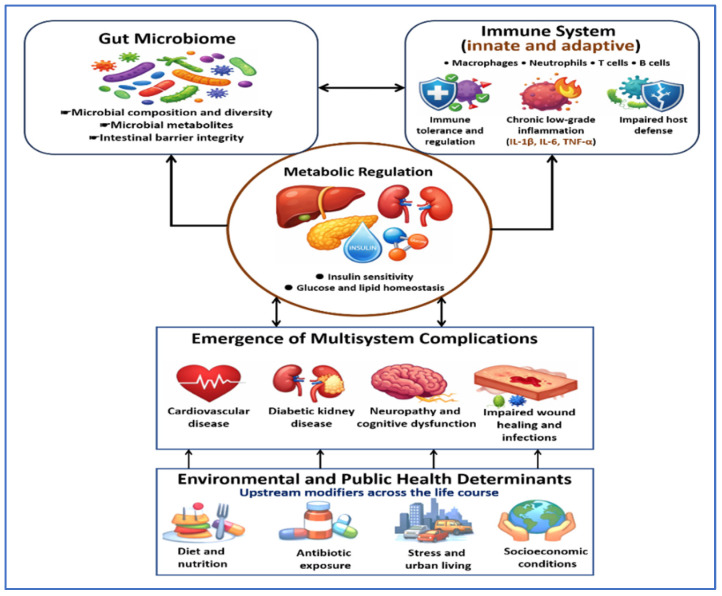
Diabetes mellitus as an interconnected microbiome immune metabolic framework. This figure demonstrates diabetes as a system-wide condition shaped by interactions among the gut microbiome, the innate and adaptive immune system, and metabolic regulation. Changes in microbial composition, microbial metabolites, and intestinal barrier integrity influence immune balance and activation. Innate and adaptive immune cells, including macrophages, neutrophils, T cells, and B cells, contribute to chronic low-grade inflammation through mediators such as Interleukin-1 beta (IL-1β), Interleukin-6 (IL-6), and Tumor necrosis factor alpha (TNF-α). These immune disturbances impair insulin sensitivity and disrupt glucose and lipid homeostasis. Over time, these processes contribute to multisystem complications, including cardiovascular disease, diabetic kidney disease, neuropathy with cognitive impairment, and impaired wound healing with increased infection risk. Environmental and public health determinants act as upstream influences that shape microbial composition, immune regulation, and metabolic health across the life course.

Figure 1 illustrates diabetes mellitus as an interconnected microbiome, immune, and metabolic network. Interactions within this network drive metabolic dysregulation and multisystem complications. Environmental and public health determinants act as upstream modifiers that shape disease risk and outcomes across the life course.

## 2. The Human Microbiome as a Metabolic Interface

Most human studies linking the gut microbiome to diabetes report associations between microbial composition and metabolic features, particularly in type 2 diabetes (T2D). These studies commonly describe reduced microbial diversity and shifts in specific taxa nonetheless do not establish causality [18]. Observational designs are suitable for identifying consistent patterns. However, they cannot determine whether microbiome changes drive metabolic disease or arise as a consequence of it [19,20].

Mechanistic insight comes primarily from experimental models. Studies using germ free animals and microbiota transfer demonstrate that microbial communities can influence host metabolism and immune regulation. Differences in physiology, immune development, and environmental exposure limit direct translation of these findings to humans [21]. This section therefore distinguishes between associations observed in human studies and mechanisms supported by controlled experimental evidence.

### 2.1. The Microbiome as a Functional Metabolic Organ

The gut microbiome contributes to metabolic regulation through continuous interaction with diet and host physiology. It participates in the breakdown of complex nutrients that cannot be fully processed by host enzymes. This process generates metabolites that act on host tissues and influence energy balance and metabolic control at both intestinal and systemic levels [22,23].

Microbial metabolites represent a central pathway linking the microbiome to host metabolism. Short-chain fatty acids are among the most well studied examples. They are produced through fermentation of dietary fiber and influence intestinal epithelial function, hormone secretion, and insulin sensitivity in peripheral tissues [23]. Through these effects, microbial metabolism links dietary intake to host energy regulation and metabolic phenotype.

Intestinal barrier integrity represents another key mechanism. An intact barrier limits the passage of microbial products into the circulation. When barrier function is impaired, microbial components enter the bloodstream and increase systemic inflammatory activity. This inflammatory state disrupts insulin signaling and contributes to metabolic dysfunction, a process commonly described as metabolic endotoxemia [22,24].

Bile acid signaling provides an additional metabolic pathway. Primary bile acids synthesized by the host are converted by gut microbes into secondary bile acids. This conversion alters bile acid pools and signaling through host receptors involved in glucose and lipid metabolism. Bile acids also influence microbial composition and activity within the intestine, indicating a reciprocal relationship between host metabolism and microbial function [24,25]. Overall, experimental and clinical evidence supports microbial metabolites, intestinal barrier function, and bile acid signaling as key mechanisms through which the gut microbiome influences metabolic regulation and diabetes risk [26].

### 2.2. Microbiome Dysbiosis in Diabetes Mellitus

A large body of research reports associations between diabetes and gut microbiome dysbiosis. In T2D, studies frequently describe reduced microbial diversity, depletion of butyrate producing organisms, and enrichment of taxa linked to inflammatory profiles. Systematic reviews consistently report higher abundance of *Escherichia* and *Shigella* and lower abundance of *Faecalibacterium prausnitzii* [18,27]. These findings are best interpreted as indicators of altered microbial function rather than disease specific microbial signatures.

Across studies, microbiome changes in diabetes align more consistently with functional pathways than with individual taxa. These pathways include altered microbial metabolite production, impaired intestinal barrier integrity, and changes in bile acid signaling. Interpretation requires caution because results vary across populations and study designs. Diet, geography, sequencing methods, analytical approaches, and medication exposure all influence observed associations. Meta analyses report reduced reproducibility when methods differ [18,28].

Medication use represents a major source of confounding. Metformin has been repeatedly associated with changes in microbial composition and function, including pathways related to short-chain fatty acid production, bile acid metabolism, and barrier integrity [29,30,31]. Microbiome patterns observed in treated individuals may therefore reflect treatment effects rather than underlying disease mechanisms [27,30,31].

Altered microbiome composition has also been reported in type 1 diabetes, particularly in children and adolescents. Studies describe differences in community structure and metabolic potential compared with non-diabetic controls, including reduced abundance of butyrate producing taxa. These findings suggest interaction between microbial ecology and immune regulation, although timing and directionality remain uncertain [32,33,34].

Studies in prediabetes provide insight into microbial changes that may precede overt disease. Differences in microbiome composition have been reported in individuals with impaired glucose tolerance, with partial overlap with T2D profiles. Substantial variation related to adiposity, diet, and phenotype limits generalization [35,36,37].

Dietary patterns and antibiotic exposure strongly influence microbiome composition and function. High fiber diets tend to support microbial fermentation and metabolite production. In contrast, low fiber diets and antibiotic exposure disrupt microbial communities and may alter immune and metabolic pathways over time [22,27,30,38,39]. These influences are particularly relevant at the population level.

Collectively, available studies favor a functional interpretation of gut microbiome dysbiosis in diabetes rather than a taxonomy-based disease signature. Available data do not support the presence of a single universal diabetic microbiome profile. Instead, observed changes reflect disruption of key pathways involving microbial metabolites, intestinal barrier function, bile acid signaling, and immune activation [40,41,42]. Immune mechanisms related to these pathways are discussed in Section 3.

Animal models have been essential for defining mechanistic links between the microbiome and metabolism. Humanized microbiome models offer partial translational insight but face limitations related to donor selection, engraftment stability, and dietary control [43]. In humans, most microbiome studies remain observational. This limits causal inference without longitudinal or interventional validation [18]. Methodological variability further complicates interpretation. Differences in sequencing platforms, analytical pipelines, and taxonomic resolution reduce cross study comparability [44]. Medication exposure, including antibiotics and commonly prescribed therapies, represents an additional source of confounding [45].

Importantly, most reported links between microbiome alterations, immune activation, and metabolic dysfunction in diabetes remain associative rather than demonstrably causal [40,46]. Experimental models provide important insight, but differences in physiology and environmental exposure limit direct translation to humans [41]. Establishing causality will require longitudinal studies and controlled interventions that modify microbial or immune pathways and assess metabolic outcomes [41,46].

Consistent with this evidence, substantial heterogeneity across populations, disease stages, diets, and treatments argues against a single diabetic microbiome pattern. Current findings support a context dependent interpretation of microbiome associations in diabetes.

Despite extensive investigation, causal links between gut microbiome features and diabetes in humans remain insufficiently defined. Reviews by Song et al. and Ruan et al. highlight how reverse causation, residual confounding, and technical variability constrain interpretation of reported associations [47,48]. Variability in sequencing platforms and data processing further limits reproducibility across studies [48]. While experimental approaches such as germ-free and microbiota transfer models provide mechanistic insight, Meijnikman et al. note that causal evidence in humans depends on rigorously controlled interventional studies [20]. These considerations underscore the need for standardized methodologies and longitudinal designs to clarify causal relevance in diabetes.

Table 1 summarizes commonly reported gut microbiome features in T2D, type 1 diabetes (T1D), and prediabetes, with emphasis on functional implications and methodological considerations.

## 3. Immune Maladaptation in Diabetes

### 3.1. Microbiome Immune Interaction and Barrier Dysfunction

The gut microbiome and the innate and adaptive immune system develop together and remain closely connected throughout life. Microbial signals support immune maturation and tolerance at mucosal surfaces. When this balance is maintained, immune activation remains controlled and intestinal barrier function is preserved. Disruption of this relationship leads to excessive or poorly regulated immune responses [51,52,53].

The intestinal barrier plays a central role in immune regulation. An intact barrier restricts the entry of microbial components into the circulation. When barrier integrity is reduced, microbial products reach the bloodstream and activate innate immune pathways that contribute to immune maladaptation [54,55,56].

Experimental studies provide mechanistic support for this process. In animal models, increased circulating lipopolysaccharide (LPS) induces inflammatory changes that promote insulin resistance and diabetes-like features. Blocking CD14-dependent signaling reduces these effects [54]. These findings introduced the concept of metabolic endotoxemia as a trigger of systemic inflammation. Differences in immune development, metabolic context, and environmental exposure limit direct translation of these models to human diabetes [57,58,59].

### 3.2. Innate Immune Activation and Adaptive Immune Imbalance in T2D

Type T2D is associated with coordinated changes in innate and adaptive immunity. Increased infiltration of macrophages into adipose tissue and pancreatic islets is linked to inflammation and metabolic impairment [60]. These macrophages often exhibit a pro-inflammatory profile, producing cytokines that interfere with insulin signaling and sustain chronic inflammation [61].

Neutrophils and other innate immune cells also show functional changes. Reduced chemotaxis and phagocytic capacity are observed in the setting of insulin resistance and persistent inflammation. Increased formation of neutrophil extracellular traps further contributes to tissue injury and inflammatory signaling [62].

Alterations in adaptive immunity reinforce this inflammatory state. Reduced numbers of regulatory T cells and expansion of pro-inflammatory T helper subsets are associated with systemic inflammation and metabolic stress [60]. These immune changes amplify inflammatory signaling and worsen metabolic dysfunction.

### 3.3. Chronic Low-Grade Inflammation as a Disease Amplifier

Chronic low-grade inflammation is a defining feature of T2D. Unlike acute inflammatory responses, this state is sustained over time and driven by nutrient excess, adipose tissue stress, and persistent innate immune activation [63,64].

Circulating inflammatory mediators reflect this ongoing immune activity. Chitinase-related proteins are associated with diabetes severity and vascular complications and indicate prolonged inflammatory stress [65,66]. These markers link gut-derived immune activation to vascular injury and metabolic deterioration [65,66,67]. Inflammasome-related mechanisms contributing to inflammatory amplification are discussed in Section 4.2.

### 3.4. Immune Dysfunction, Infection Risk, and Impaired Healing

Immune maladaptation in diabetes has clear clinical consequences. Reviews of diabetic foot disease and chronic wounds describe impaired early immune responses. These include reduced neutrophil activity, delayed microbial clearance, and prolonged inflammation. Such defects increase infection risk and delay tissue repair [68,69,70].

Inflammatory responses in diabetic wounds often persist beyond appropriate time frames. This delay interferes with tissue remodeling and promotes chronic non-healing wounds [70,71]. These outcomes reflect systemic immune dysfunction rather than isolated local complications. They support the need to consider immune health alongside metabolic control in clinical care [68,72].

Dietary patterns with lower inflammatory potential have practical relevance. Diets rich in fiber and minimally processed foods are associated with favorable microbial profiles and reduced systemic inflammation [73,74]. Increased intake of fermentable fiber supports barrier integrity through enhanced short-chain fatty acid production, which contributes to mucosal defense [23,75]. By limiting microbial translocation, strategies that preserve barrier function may reduce chronic immune activation and improve host defense. Clinical validation of these approaches remains limited [41,55,76,77].

### 3.5. Context Dependence and Limitations of Immune Mechanisms

Although this review emphasizes T2D, immune mechanisms differ across diabetes phenotypes. In T1D, immune dysfunction arises from loss of immune tolerance and autoimmune destruction of pancreatic beta cells. Early-life microbial exposure influences immune development and disease risk [78,79]. In contrast, immune dysfunction in T2D reflects chronic inflammation driven by nutrient excess and metabolic stress [80]. Despite these differences, both conditions involve interactions among microbial composition, immune regulation, and metabolic state [41].

Evidence on immune pathways in diabetes is derived largely from experimental systems and associative human studies. Reviews by Mauvais and colleagues note that animal and in vitro models do not fully capture the complexity of human immune responses or disease progression [81]. Immune activity varies by diabetes type, disease stage, age, and treatment exposure, which limits generalization across populations [80,81,82]. Immune pathways should therefore be viewed as contributing processes rather than universal drivers of disease [83]. Longitudinal human studies are required to clarify timing, directionality, and clinical relevance of immune alterations in diabetes [20].

## 4. Metabolic Dysfunction as an Emergent Systemic Outcome

### 4.1. Microbial Metabolites and Host Metabolism

Metabolic regulation is influenced by signals that originate in the gut. Among these signals, microbiome-derived metabolites represent a direct link between microbial activity and host metabolism. These metabolites affect insulin sensitivity, energy balance, and inflammatory tone through specific host signaling pathways. This section focuses on their metabolic relevance, while structural features of the microbiome are addressed in Section 2.

Evidence from systematic reviews and human intervention studies shows that changes in microbial metabolite profiles are associated with metabolic outcomes relevant to diabetes [84]. Responses vary widely between individuals. This variation reflects differences in diet, baseline microbial composition, genetic background, and medication exposure, including commonly prescribed diabetes therapies [85,86,87].

Short-chain fatty acids provide a clear example. Higher levels after dietary or microbial interventions are associated with lower fasting insulin and improved insulin sensitivity. These findings support a regulatory role rather than simple energy provision [88]. Microbial metabolites also influence gut hormone release. Hormones such as glucagon-like peptide-1 (GLP-1) and peptide YY link microbial activity to appetite regulation and glucose control, providing a pathway between the gut and systemic metabolism [89].

Disruption of intestinal barrier function represents another pathway linking microbial signals to metabolic dysfunction. Experimental studies show that increased passage of microbial products into the circulation promotes inflammation and insulin resistance. Blocking LPS signaling reduces these effects [54]. This pathway connects microbial activity to metabolic impairment primarily through immune mechanisms discussed in Section 3.

### 4.2. Inflammation Metabolism Feedback Loops

**Figure 2:**
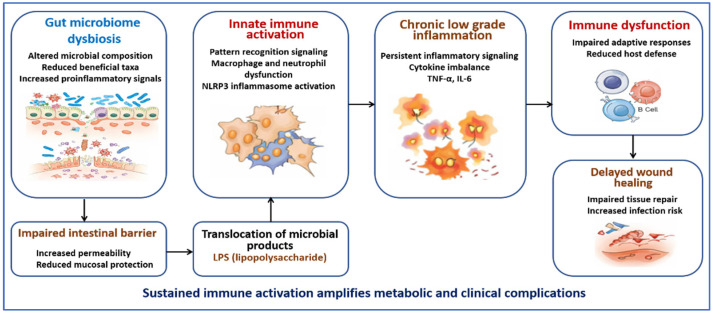
Pathways linking gut microbiome dysbiosis to immune maladaptation in diabetes. This figure shows how alterations in gut microbial composition and loss of intestinal barrier integrity increase permeability and allow microbial products, including LPS, to enter the circulation. These signals activate innate immune pathways through pattern recognition receptors and disrupt macrophage and neutrophil function. Activation of the NOD-like receptor family pyrin domain containing 3 (NLRP3) inflammasome further amplifies inflammatory signaling. Sustained innate immune activation promotes chronic low-grade inflammation mediated by cytokines such as TNF-α and IL-6. Over time, this inflammatory state impairs both innate and adaptive immune responses, reduces host defense, and delays wound healing.

Sustained inflammation and metabolic dysfunction reinforce each other in diabetes. Microbial products and nutrient excess activate innate immune pathways. Inflammatory signaling then interferes with insulin action in metabolic tissues. Over time, this interaction amplifies both metabolic stress and immune activation (Figure 2) [54].

The NLRP3 inflammasome represents a key molecular contributor to this process. Clinical and translational studies associate its activation with cytokine release, insulin resistance, and vascular complications in T2D [90,91]. These findings indicate that inflammatory activation can persist independently of glycemic control and contribute to progressive metabolic impairment.

Downstream cytokines further propagate dysfunction. IL-1β and IL-18 promote beta cell stress and impair insulin secretion. TNF-α and IL-6 disrupt insulin signaling and endothelial function [92,93]. Persistent inflammatory signaling also interferes with tissue repair. At the cellular level, immune cells undergo metabolic shifts during chronic inflammation and nutrient excess. These changes reinforce both insulin resistance and inflammatory persistence [94].

From a clinical perspective, these pathways inform risk assessment and adjunctive strategies rather than replacing glucose-lowering therapy. They help explain why glucose control alone may not fully reduce risk when upstream drivers such as dysbiosis, excess adiposity, and barrier dysfunction remain present. This supports approaches that address metabolic regulation and inflammatory burden together, while acknowledging current limits of clinical application [37,90]. Key mediators are summarized in Table 2.

### 4.3. Reframing Hyperglycemia

Hyperglycemia remains central to the diagnosis and monitoring of diabetes; however, it does not fully capture the biological complexity of the disease. Evidence discussed in earlier sections indicates that microbial signals and innate immune activation influence insulin sensitivity and inflammatory tone. These processes interact with metabolic regulation and may precede the onset of clinical diabetes or persist even when glycemic control improves [54,88].

Clinically, this means that while hyperglycemia remains central to diagnosis, it does not fully account for the development of diabetic complications. Adopting a broader perspective encourages consideration of inflammation, intestinal barrier integrity, diet quality, and microbial metabolism alongside glucose management. At the same time, it recognizes the need for more robust clinical evidence before these concepts can be translated into routine practice [88,95].

**Table 2:** Microbial and immune related mediators contributing to metabolic dysfunction in diabetes.

Mediator or Pathway	Primary Source	Metabolic Effects Reported	Clinical Relevance	Predominant Level of Evidence	Key References
Short-chain fatty acids	Microbial fermentation of dietary fiber	Lower fasting insulin and improved insulin sensitivity after intervention	Supports dietary strategies that increase fermentable fiber intake	Meta-analyses and human intervention studies	[88]
Propionate and gut hormone signaling	Microbial fermentation with colonic delivery	Increased GLP-1 and peptide YY release. Reduced energy intake	Links microbial metabolites to appetite control and metabolic regulation	Experimental and human clinical studies	[96]
Bile acid modification and receptor signaling	Host bile acid synthesis with microbial conversion	Regulation of glucose and lipid metabolism through FXR and TGR5 signaling	Supports bile acid and microbiome targeted interventions	Experimental and translational studies	[97,98]
Microbial products and metabolic endotoxemia	Barrier dysfunction and microbial translocation	Increased inflammation and insulin resistance in experimental models	Highlights the importance of intestinal barrier integrity	Experimental animal studies	[54]
NLRP3 inflammasome activation	Innate immune activation under metabolic stress	Chronic inflammation associated with insulin resistance	Supports inflammation focused therapeutic strategies	Experimental and translational studies	[99,100]
IL-1 pathway inhibition	Clinical anti-inflammatory intervention	Improved glycemic control in T2D	Clinical proof of concept for inflammation targeted therapy	Clinical randomized trials	[101]

## 5. Multisystem Complications as a Failure of Organ Crosstalk

### 5.1. Cardiovascular Complications

Cardiovascular disease remains the leading cause of illness and death in people with diabetes. Risk reflects the combined effects of chronic inflammation, endothelial dysfunction, lipid abnormalities, and sustained metabolic stress. These processes act together rather than independently and indicate disrupted communication across metabolic, immune, and vascular systems [102,103].

Gut-derived metabolites provide evidence of this cross-organ disturbance. Trimethylamine N-oxide (TMAO) is generated from dietary precursors through microbial activity followed by hepatic metabolism. Meta-analyses and narrative reviews report associations between higher circulating levels and cardiovascular disease, as well as cardiometabolic risk markers in diabetes. These findings support TMAO as a marker of gut-related cardiovascular risk, while causality and therapeutic relevance remain under investigation [102,103,104].

Traditional cardiovascular risk assessment focuses largely on lipid measures. Gut-derived markers reflect inflammatory and metabolic processes not captured by standard lipid profiles. They therefore complement rather than replace established risk models [105,106,107]. Randomized trials that directly target gut-related cardiovascular pathways in diabetes remain limited. This gap highlights the need for interventional studies to determine whether modifying these pathways provides benefit beyond conventional lipid-lowering strategies [107,108,109].

From a clinical standpoint, cardiovascular risk in diabetes reflects failure of coordinated organ responses rather than a single dominant factor. This supports prevention strategies that address diet quality, inflammatory burden, and metabolic control together [102,103].

### 5.2. Diabetic Kidney Disease

Diabetic kidney disease is a major cause of chronic kidney failure worldwide. While hyperglycemia and hypertension are central drivers, innate immune activation and chronic inflammation modify disease progression. Increasing evidence points to bidirectional communication between the gut and the kidney as a contributing factor rather than a secondary effect [110,111].

Two linked processes characterize this interaction. Reduced kidney function promotes gut barrier disruption and microbial imbalance through uremia and systemic inflammation. At the same time, altered gut microbial activity increases production of uremic toxins and inflammatory compounds that enter the circulation and contribute to renal injury. Reviews identify these reciprocal pathways as important mediators of kidney disease progression in diabetes [110,111,112].

Inflammation-related biomarkers provide further insight. Chitinase-related proteins correlate with renal dysfunction and progression of diabetic complications. These markers reflect sustained innate immune activation across organ systems [65,66].

From a clinical and preventive standpoint, these observations have practical implications. Nutritional approaches that increase dietary fiber are being explored as strategies to reduce circulating toxins and inflammatory burden. Although evidence is still emerging, this framework supports care approaches that link nutrition, inflammation, and kidney outcome [110,111].

### 5.3. Neuropathy and Cognitive Dysfunction

Diabetic neuropathy and cognitive impairment reflect the effects of diabetes on the nervous system through systemic processes. Neuroinflammation, vascular injury, and metabolic stress contribute to peripheral nerve damage and cognitive decline. These processes intersect with gut-derived signals through gut and brain communication pathways [113,114].

Reviews examining diabetes and cognition describe interactions involving innate immune activation, inflammatory mediators, microbial metabolites, and hormonal signaling. Elevated inflammatory markers, including chitinase-related proteins, are associated with neurovascular and inflammatory changes in diabetes and may contribute to neurologic complications [65,66]. Most evidence remains associative, but findings align with a broader pattern of multisystem dysfunction [113,114,115].

From a clinical perspective, these findings converge on a consistent message. Neurologic complications in diabetes cannot be explained by glucose levels alone. They reflect systemic disturbances involving inflammation, vascular health, and metabolic regulation. This supports prevention strategies that extend beyond glycemic control [113,115].

### 5.4. Impaired Wound Healing and Infection Risk

Impaired wound healing and increased susceptibility to infection are among the most consequential complications of diabetes. Diabetic wounds often persist due to blunted early immune responses and prolonged inflammation. Vascular impairment and reduced tissue oxygen delivery further limit effective repair. Reviews of diabetic wound biology describe how these factors increase the risk of chronic wounds and infection [68,70].

Immune dysfunction affects multiple components of host defense. Reviews report impaired neutrophil migration and phagocytosis, reduced antimicrobial activity, and altered macrophage function in diabetic wounds. These defects link systemic immune disturbance to local tissue failure, delaying repair and reducing pathogen clearance [68,69,116].

**Figure 3:**
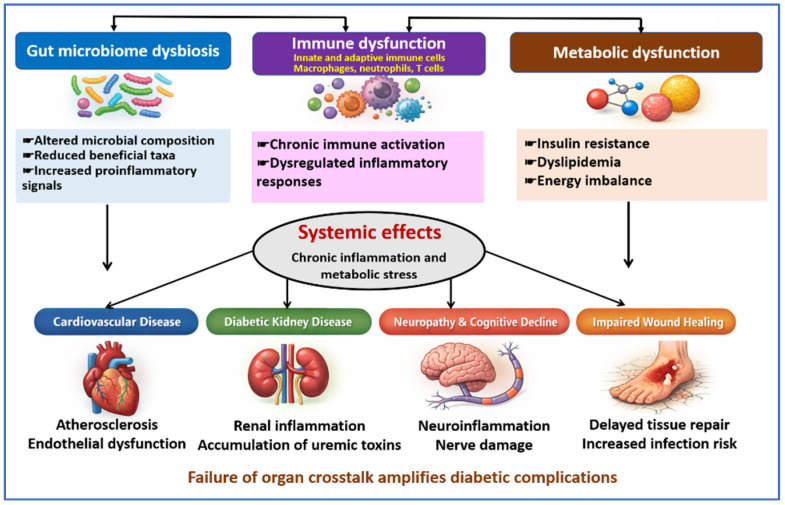
Multiorgan pathways linking microbiome, immune, and metabolic dysfunction to diabetic complications. This figure illustrates how disturbances in gut microbial activity, immune function involving innate and adaptive responses, and metabolic regulation act as upstream influences in diabetes. These processes interact over time and give rise to shared systemic effects marked by persistent inflammation and metabolic stress. Directional arrows indicate progression from upstream biological disturbances to downstream organ involvement. Impaired communication among organs contributes to cardiovascular disease, diabetic kidney disease, neuropathy with cognitive impairment, and delayed wound healing with increased susceptibility to infection.

These complications carry substantial public health consequences. They increase hospital admissions, antibiotic exposure, healthcare costs, and long-term disability. This underscores the need for care strategies that address metabolic control, immune function, vascular health, and infection prevention in parallel [68,70]. Figure 3 summarizes how combined disturbances in microbial activity, immune regulation, and metabolic control contribute to cardiovascular, renal, neurologic, and wound-related complications in diabetes.

## 6. Environmental and Public Health Determinants

### 6.1. Dietary Transitions and Microbial Loss

Dietary patterns in many populations have shifted toward higher intake of ultra-processed foods. Meta-analyses report a dose response association between ultra-processed food consumption and risk of T2D, with higher intake linked to greater risk [117]. At the individual level, dietary counseling can prioritize reducing ultra-processed foods and increasing dietary fiber. These approaches are associated with improved metabolic regulation and lower inflammatory markers in observational studies and dietary intervention trials [118,119].

Higher dietary fiber intake is consistently associated with favorable metabolic profiles. Systematic reviews show that increased short-chain fatty acid production following dietary intervention is linked to lower fasting insulin and improved insulin sensitivity [88,120]. At the population level, policies that improve access to fiber-rich foods may support metabolic health and reduce inflammatory burden [119,121,122]. These findings support prevention strategies that emphasize diet quality, including lower intake of ultra-processed foods and higher consumption of fiber-rich foods [123]. Observational dietary studies remain subject to residual confounding related to socioeconomic status, lifestyle, and co-occurring health behaviors [124,125].

### 6.2. Antibiotic Exposure and Immune Imprinting

Antibiotic exposure alters gut microbial composition and may have lasting effects when exposure is repeated or occurs early in life. Meta-analyses report that antibiotic use during infancy is associated with a modest increase in the risk of childhood overweight and obesity, a major upstream risk factor for T2D [126,127]. Early disruption of microbial development may influence immune maturation and long-term inflammatory regulation, consistent with life-course models of metabolic risk [128,129].

In adults, observational studies link long-term antibiotic use with increased risk of T2D, although causality cannot be established [130]. Prescribing decisions at the individual level may influence personal metabolic risk. At the population level, stewardship programs limit cumulative exposure and reduce unnecessary disruption of microbial communities [131]. These findings support antibiotic stewardship as a public health priority that balances infection treatment with long-term metabolic health [126,130].

### 6.3. Urbanization, Stress, and Circadian Disruption

Urban living is associated with behaviors that influence metabolic health, including irregular sleep patterns and disrupted daily schedules. Meta-analyses of cohort studies show that night shift work is associated with higher risk of T2D [132]. Circadian disruption affects immune signaling and daily microbial rhythms, providing a biological link between urban exposures and metabolic risk [133,134,135].

Sleep duration shows a consistent U-shaped association with diabetes risk. Both short and long sleep durations are linked to higher incidence compared with seven to eight hours per night [136]. Evidence linking psychosocial stress to diabetes risk is less consistent. Observed associations appear to depend on context and often coexist with sleep disruption and socioeconomic stressors [137,138]. Residual confounding remains a major limitation in observational studies of stress and metabolic outcomes [135].

### 6.4. Global Disparities and Health Inequities

Diabetes risk and outcomes are strongly shaped by social and economic conditions. A scientific review from the American Diabetes Association (ADA) highlights how social determinants influence prevention, diagnosis, self-management, and complications through pathways that include food access, education, chronic stress, and access to healthcare [139]. These factors also shape biological vulnerability through sustained nutritional and psychosocial strain [140,141].

A substantial proportion of diabetes cases worldwide remain undiagnosed. The International Diabetes Federation (IDF) reports a large global burden of undiagnosed diabetes and emphasizes the economic and clinical impact of delayed diagnosis, which increases complication burden at presentation [2]. Food insecurity represents a key pathway linking disadvantage to challenges in diabetes care. It is associated with poorer diet quality and barriers to consistent self-management, although associations with glycemic measures vary across populations and study designs [142,143,144].

These inequities place significant strain on health systems. Complications increase hospital admissions, costs, and long-term disability. Effective responses require coordinated approaches that combine clinical care, nutrition support, community resources, and policy action [2,139]. At a global level, these findings support prioritizing upstream social and environmental drivers alongside biological research [145,146]. Table 3 summarizes key environmental and public health factors influencing the microbiome, immune, and metabolic axis.

### 6.5. Evidence for Policies and Interventions

Population-level policies provide evidence for prevention beyond individual counseling. Taxes on sugar-sweetened beverages have been widely evaluated as measures to reduce sugar intake. Systematic reviews of real-world evaluations show that these taxes increase prices and reduce sales and purchases of taxed beverages across multiple countries [148]. These interventions complement, rather than replace, individual dietary guidance [149,150].

Natural experiments in Mexico and several U.S. cities demonstrate sustained reductions in purchases after tax implementation, with larger effects among high-consumption groups [151,152]. Modeling studies based on observed consumption changes estimate that a 20 percent tax could reduce obesity prevalence and lower future incidence of T2D [153]. These estimates rely on modeled assumptions and observational data and remain subject to uncertainty and residual confounding [122,154,155].

Antibiotic stewardship programs aim to reduce unnecessary use and limit long-term microbial disruption. A Cochrane systematic review shows that stewardship interventions reduce antibiotic prescribing without increasing adverse clinical outcomes [156]. At the individual level, stewardship supports preservation of immune and microbial function. At the population level, it reduces cumulative exposure that may influence metabolic risk across the life course [128,131,157,158].

Observational cohort studies also link repeated or early-life antibiotic exposure to persistent microbial alterations and increased risk of overweight and metabolic dysregulation [159]. These associations suggest long-term effects of early exposures, although causality remains uncertain [128,129,160].

Across the life course, environmental exposures and public health policies meaningfully influence diabetes risk by shaping diet quality, microbial development, immune regulation, and access to care [41]. Individual-level counseling and population-level interventions act through complementary pathways. Together, they position prevention as a central strategy to reduce long-term disease burden and health system costs, particularly in high-risk populations [139,161].

## 7. Clinical Implications and Emerging Care Models

### 7.1. Limitations of Current Therapeutic Paradigms

Glycemic control remains essential for diagnosis, monitoring, and treatment decisions in diabetes. Sustained glucose lowering reduces the risk of several microvascular complications. However, many individuals develop complications despite achieving recommended glycemic targets. This pattern reflects the systemic nature of diabetes, which involves inflammatory, vascular, and organ-level dysfunction beyond hyperglycemia alone [162,163,164].

Large clinical trials illustrate both the benefits and limits of intensive glucose control. In the Action in Diabetes and Vascular Disease: Preterax and Diamicron Modified Release Controlled Evaluation trial, intensive treatment reduced a composite outcome driven mainly by microvascular event [162,165]. In contrast, the Action to Control Cardiovascular Risk in Diabetes trial was stopped early because of increased mortality in the intensive treatment group [166,167]. These findings support a cautious approach. More intensive glucose lowering is not appropriate for all patients, particularly when hypoglycemia risk and comorbidity burden are high [168].

Long-term follow-up studies further show that benefits and harms emerge over different time scales. Follow-up analyses from the Veterans Affairs Diabetes Trial reported modest long-term cardiovascular effects and sustained renal benefit with intensive glucose control [164,169]. Overall, these trials indicate that glucose-focused treatment alone may be insufficient when parallel inflammatory, vascular, and organ-level processes remain active [170]. They support individualized glycemic targets within broader care strategies.

In routine care, fragmentation remains a major limitation. Patients often receive separate guidance for glucose control, weight, blood pressure, lipids, sleep, and stress. A systems-oriented approach addresses this gap by positioning glycemia as one clinical marker among several contributors to disease progression, rather than as an isolated treatment goal [41,171,172].

### 7.2. Microbiome Informed Prevention and Treatment

Microbiome-informed care should emphasize interventions with strong evidence for diabetes prevention and cardiometabolic risk reduction. Lifestyle intervention provides the clearest example. In the Diabetes Prevention Program (DPP), intensive lifestyle modification reduced incident T2D by 58% over a mean follow-up of 2.8 years, exceeding the effect of metformin [173]. Dietary change was central to this intervention and aligns with principles such as increasing minimally processed foods and dietary fiber [174,175,176].

Mediterranean dietary patterns are supported by additional evidence. In the PREDIMED study, Mediterranean diet interventions were associated with lower incidence of T2D among older adults at high cardiovascular risk [177]. These effects occurred with minimal weight change, suggesting that dietary quality influences metabolic risk through pathways beyond weight alone.

Probiotics are widely used and increasingly discussed in clinical practice. Meta-analyses of randomized trials report small improvements in glycemic markers, including HbA1c and fasting glucose, in T2D. Effect size and certainty vary across studies [178,179]. These findings support a cautious interpretation. Probiotics may provide modest benefit for selected individuals but should not be considered standalone therapy. Response depends on baseline microbial features, diet, medication exposure, and disease stage [179,180].

Fecal microbiota transplantation has provided proof of concept that altering gut microbial communities can influence insulin sensitivity in humans. A controlled study reported improved insulin sensitivity after microbiome transfer from lean donors in individuals with metabolic syndrome [181]. Subsequent studies in T2D show mixed results, with some reporting no meaningful improvement in insulin sensitivity or HbA1c [182,183]. This variability highlights an important limitation. Diabetes does not represent a single biological state, and durable benefit likely depends on alignment with host characteristics, diet, and lifestyle context.

Several evidence gaps remain. Many microbiome studies are short in duration, small in size, and methodologically heterogeneous. Patient-centered clinical outcomes are often not assessed. Microbiome testing is not yet standardized for routine clinical use. At present, the most reliable applications of microbiome-informed care lie in evidence-based lifestyle strategies rather than adjunctive microbial therapies.

### 7.3. Multidisciplinary and Patient-Centered Care

A systems-based view of diabetes aligns with team-based care models. Effective management requires attention to metabolic targets, infection risk, wound healing, nutrition quality, and social context. It also depends on coordinated communication across clinical disciplines. The ADA Standards of Care emphasize diabetes self-management education and support and highlight the importance of structured care processes and attention to social determinants of health [172,184].

Diabetes self-management education and support improve outcomes in T2D. Systematic reviews report reductions in HbA1c following education and support interventions, although effect size varies by program design and setting [185,186]. These programs demonstrate how coordinated care can integrate medical management, nutrition counseling, behavioral support, and follow-up within a unified pathway.

Patient-centered care also requires recognition of barriers beyond the clinic. Food insecurity, limited access to healthy foods, and constraints on sleep and physical activity often shape what is feasible for individuals. When these barriers are unaddressed, treatment may rely on medication intensification while upstream drivers persist. Multidisciplinary care models allow lifestyle and prevention strategies to be adapted to real-world contexts, supporting sustained engagement and adherence [172,185].

**Figure 4:**
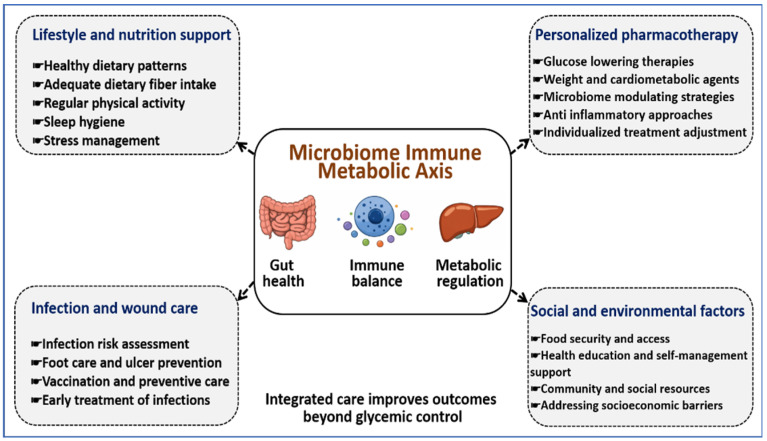
Clinical care approach addressing microbiome, immune, and metabolic influences in diabetes. This figure presents a patient-centered care approach that combines lifestyle and nutrition support, individualized pharmacotherapy, infection and wound risk management, and attention to social and environmental factors. These elements act on gut health, immune balance, and metabolic regulation as interconnected contributors to disease progression. Arrows indicate coordinated interactions among care components across clinical and community settings. The figure emphasizes how addressing biological processes alongside contextual factors can improve diabetes outcomes beyond glucose control alone.

Figure 4 presents a coordinated clinical care approach that addresses metabolic control, infection and wound risk, nutrition quality, and social and environmental determinants through structured, patient-centered care.

## 8. Future Directions and Research Priorities

### 8.1. Longitudinal and Interventional Study Designs

Meaningful progress in this field will require study designs capable of distinguishing causal mechanisms from downstream consequences. Cross-sectional microbiome studies in diabetes often report inconsistent taxa and pathways across populations. This reflects true biological diversity and differences in diet, geography, and medication use. These factors limit translation when disease-related signals cannot be separated from contextual influences [187,188].

Longitudinal cohorts are therefore a priority. Studies that follow individuals from normoglycemia through prediabetes and diabetes can identify early changes that precede metabolic deterioration. Such designs also support prevention by identifying modifiable exposures before disease onset. Parallel measurement of host and microbial features over time provides insight into dynamic changes that single time-point studies cannot capture [189]. Long-term follow-up, analytical cost, and limited infrastructure remain barriers, particularly in low-resource settings, which may constrain generalizability.

Interventional trials are equally important. These studies test whether modifying microbial features alters metabolic outcomes. Early controlled trials reported improved insulin sensitivity after microbiome transfer from lean donors in individuals with metabolic syndrome [181]. Later trials showed variable responses influenced by baseline microbial features and donor characteristics [183,190]. More recent double-blind studies suggest that combining dietary change with microbiome-targeted approaches may be necessary to achieve sustained effects [191]. These findings support testing interventions within lifestyle contexts rather than as isolated biological treatments.

Methodological consistency remains a challenge. The Strengthening the Organization and Reporting of Microbiome Studies checklist provides guidance for transparent reporting of study design, laboratory methods, data processing, and confounders. Wider adoption would improve reproducibility and strengthen comparative evidence [192].

### 8.2. Biomarker Development and Personalized Risk Assessment

Precision approaches require biomarkers that add value beyond established clinical predictors. Microbiome-based risk indicators represent one possible direction. A prospective metagenomic study reported that disrupted daily variation in gut microbial patterns was associated with T2D and improved risk prediction when combined with body mass index [193].

For biomarkers to be useful, they must perform consistently across populations and settings. This requires replication, careful control of confounders such as medication use, and standardized analytical approaches. Large multi-cohort studies that combine microbial data with proteomic or metabolomic measures suggest that layered signatures may provide more stable signals than individual microbial features alone [187]. Such approaches may also help identify individuals and communities most likely to benefit from targeted health promotion strategies. At the same time, the growing availability of commercial microbiome tests raises concern about premature clinical use without adequate validation or demonstrated benefit [194].

Host-derived markers remain central to risk assessment. Precision medicine in diabetes extends beyond microbial measures. The Precision Medicine in Diabetes Initiative of the ADA and the European Association for the Study of Diabetes defines precision care as the integration of clinical phenotypes and biomarkers to improve diagnosis, prevention, and treatment, while emphasizing the need for strong evidence before implementation [194,195].

A practical near-term goal is combined risk assessment. Models that integrate established clinical markers with microbiome-informed signals should be evaluated by their ability to improve prediction, guide prevention decisions, or identify individuals at higher risk of complications. Without such added value, biomarker development is unlikely to influence care.

Several immune and inflammatory markers show emerging relevance. High-sensitivity C-reactive protein is consistently associated with insulin resistance, cardiovascular risk, and incident T2D in prospective studies, although it lacks disease specificity [196,197]. Markers related to the IL-1 pathway, including IL-1β and IL-18, are linked to beta cell stress and inflammatory activity and represent some of the most advanced immune targets in translational research [80,198]. IL-6 and TNF-α are also associated with insulin resistance and vascular dysfunction, but variability across populations limits their use as standalone markers [83,199]. In contrast, most microbiome-derived inflammatory indicators remain exploratory and lack longitudinal validation [46,200]. Further work is needed to determine whether these measures improve prediction beyond established clinical risk factors [40,201].

### 8.3. Implementation Science and Clinical Validation

Strong evidence does not guarantee clinical uptake. Microbiome-informed strategies must be feasible, acceptable, and scalable within real healthcare systems. Implementation science provides tools to evaluate delivery, adoption, and sustainability. A recent systematic review of DPP implementation reported wide variation in terminology and reporting and emphasized the need for standardized methods to assess implementation outcomes [202].

These challenges are particularly relevant for precision prevention. A recent review of precision prevention in T2D highlights how social and structural conditions shape every stage of prevention and influence who benefits from new strategies [203]. Constraints related to cost, workforce capacity, and infrastructure are especially important in low-resource settings and may widen existing inequities if not addressed.

### 8.4. Policy Implications and Preventive Strategies

Population-level prevention remains essential because many drivers of diabetes operate at scale. The World Health Organization (WHO) identifies taxes on sugar-sweetened beverages as one policy option to reduce consumption and improve diet quality and provides guidance for implementation [204].

Broader policy packages are also required. The WHO best buys framework summarizes cost-effective interventions to reduce noncommunicable disease risk factors, including unhealthy diet and physical inactivity [205,206]. From a systems perspective, policy success should be evaluated using multiple outcomes, including dietary quality, inflammatory burden, equity, healthcare use, and long-term complication risk, rather than incidence alone.

Future progress will depend on coordinated efforts. These include rigorous longitudinal and interventional studies, cautious and evidence-based biomarker development, implementation research that supports equitable scale-up, and prevention policies evaluated using multidimensional health outcomes rather than short-term behavioral change alone [194,205].

## 9. Conclusions

Diabetes mellitus extends beyond abnormal blood glucose levels. It is a systemic condition shaped by interactions among gut microbial activity, immune function, and metabolic regulation. This view helps explain why individuals with similar glycemic profiles can follow different disease courses and face different risks of complications. Disturbances in microbial balance and immune regulation contribute to persistent inflammation and metabolic stress. These processes are often not fully addressed by glucose-focused treatment alone. Environmental and social conditions further influence disease progression by shaping diet quality, medication exposure, sleep patterns, and access to care. This review synthesizes evidence across microbial, immune, metabolic, and public health domains to clarify how diabetes develops and progresses as a multisystem disorder. Recognizing these interactions supports prevention strategies that extend beyond glycemic targets, informs more comprehensive clinical care, and highlights priorities for future research and population-level action aimed at improving long-term outcomes in diabetes.

## Figures and Tables

**Table 1:** Reported gut microbiome alterations across diabetes phenotypes.

Diabetes Phenotype	Commonly Reported Microbiome Features	Functional Implications	Key Considerations and Limitations	Key References
T2D	Reduced microbial diversity. Increased abundance of *Escherichia* and *Shigella*. Reduced abundance of *Faecalibacterium prausnitzii*.	Reduced butyrate production. Impaired intestinal barrier function. Increased inflammatory tone linked to insulin resistance.	Strong influence of diet and medication exposure. Metformin is a major confounder in observational studies.	[18,49]
T1D	Altered microbiome development in children at risk. Functional shifts reported before autoimmunity onset.	Possible effects on immune tolerance, barrier integrity, and inflammatory signaling.	Evidence strongest in pediatric cohorts. Timing and causality remain uncertain.	[50,51]
Prediabetes	Intermediate microbiome changes. Reduced abundance of butyrate producing taxa in some cohorts.	Early metabolic and inflammatory alterations associated with insulin resistance.	High variability across studies. Strong influence of phenotype definition, adiposity, and diet.	[35,36]

**Table 3:** Environmental and public health factors influencing the microbiome, immune, and metabolic axis.

Environmental or Public Health Factor	Typical Exposure Pattern	Links to Microbiome, Immune, and Metabolic Pathways	Public Health and Clinical Implications	Key References
Ultra-processed foods	High proportion of daily energy intake	Higher intake increases T2D risk in a dose-dependent manner, likely via microbiome and inflammatory changes.	Supports policies to improve food environments and promote minimally processed diets.	[117]
Low dietary fiber intake	Low intake of whole grains, legumes, fruits, and vegetables.	Reduced microbial fermentation and SCFA production, linked to insulin resistance and metabolic dysfunction.	Supports dietary counseling and fiber-focused prevention strategies.	[88]
Early life antibiotic exposure	Antibiotic use during infancy, often repeated	Disrupted microbiome development increases childhood obesity risk, raising later T2D risk.	Supports antibiotic stewardship during early life	[126]
Long term antibiotic exposure	Repeated or prolonged antibiotic use in adulthood.	Observed associations with higher T2D risk, consistent with cumulative microbiome disruption.	Reinforces cautious antibiotic prescribing and awareness of long-term risks.	[130,147]
Night shift work	Rotating or permanent night work schedules	Circadian disruption is associated with higher T2D incidence in cohort meta-analyses.	Supports workplace and clinical strategies to reduce circadian misalignment.	[132]
Sleep duration extremes	Habitual short or long sleep duration	U-shaped association with T2D risk, reflecting hormonal and inflammatory dysregulation.	Supports routine sleep assessment in diabetes prevention	[136]
Socioeconomic disadvantage	Limited access to resources, education, and healthcare	Linked to higher diabetes risk and complications via poorer diet quality, greater stress, and limited access to care.	Supports equity focused health and social policies	[139]
Food insecurity	Unstable access to sufficient and nutritious food	Associated with barriers to effective diabetes self-management and prevention.	Supports screening, nutrition assistance, and integrated care programs	[142]
Undiagnosed diabetes	Limited access to screening and preventive services	Delayed diagnosis is linked to greater complication burden at presentation.	Supports population-level screening and early detection strategies.	[2,16]

## Data Availability

No new data were created or analyzed in this study.
